# Supplementary material for: Potential for Applying Continuous Directed Evolution to Plant Enzymes: An Exploratory Study
Source: Life (Basel). 2020 Sep 5;10(9):179. doi: 10.3390/life10090179 (PMC7555113; doi:10.3390/life10090179)
Supplement: Supplementary file 1 [file life-10-00179-s001.pdf]

life

MDPI

SUPPLEMENTARY INFORMATION

Table S1. Primers used in the present work.

No. of primer	Name	Sequence 5´- 3´
1	ScTHI4_NsiI_Fw	CGATATGCATTCTGCTACCTCTACTGCTA
2	ScTHI4_SphI_Rv	ATCGGCATGCCTAAGCAGCAAAGTGTTTC
3	TP DNAP1_AREC318_WT_FW	GGTAGACCAAACCATGACTTTGG
4	TP DNAP1_AREC318_WT_Rv	CCACAATGAGTTCATGATGAT
5	TP DNAP1_AREC611_ep_Rv	CACAACAATTTATCAAATTCACATTCTAATTCAG
6	TP DNAP1_AREC633_ep_Rv	GATCAACATGAAGTCTGTATCGTT
7	TvThi4F1	TAGCGAGCGAAGTTGTTATTAGCG
8	TvThi4R1	AAACCGCTAATAACAACTTCGCTC
9	TvThi4F2	TAGCGATGTGGCGATTGTGGGTGG
10	TvThiR2	AAACCCACCCACAATCGCCACATC
11	TvThi4F3	TAGCGACGTTCGAAGATCGCCACA
12	TvThi4R3	AAACTGTGGCGATCTTCGAACGTC
13	TvThi4F4	TAGCCGAGATCGTGGTTCAGGAGA
14	TvThi4R4	AAACTCTCCTGAACCACGATCTCG
15	TvThi4F5	TAGCATTTAAGCCGGGTTACTATC
16	TvThi4R5	AAACGATAGTAACCCGGCTTAAAT
17	TvThi4F6	TAGCGCGGGTGCGACCGTGTTTAA
18	TvThi4R6	AAACTTAAACACGGTCGCACCCGC
19	TvThi4F7	TAGCAACGGTCAATACCGTGTGTG
20	TvThi4R7	AAACCACACACGGTATTGACCGTT
21	TvThi4F8	TAGCTGGTTGGCGAGAAACCGCTG
22	TvThi4R8	AAACCAGCGGTTTCTCGCCAACCA
23	TvThi4F9	TAGCGAACAGCAAAGAAGTGTTCC
24	TvThi4R9	AAACGGAACACTTCTTTGCTGTTC
25	TvThi4F10	TAGCCGGTAGCCACCGTATGGGTC
26	TvThi4R10	AAACGACCCATACGGTGGCTACCG
27	TvThi4F11	TAGCAAAGGTGGCGGAGGAGATTG
28	TvThi4R11	AAACCAATCTCCTCCGCCACCTTT
29	1944ThermThi4F1F	TAGCGGTTATTACCGCGAAATATG
30	1945ThermThi4F1R	AAACCATATTTCGCGGTAATAACC
31	1946ThermThi4F2F	TAGCGGTTAGCACCCTGCAGCGTA
32	1947ThermThi4R2R	AAACTACGCTGCAGGGTGCTAACC

Table S2. Compositions of buffers used in the protoplast fusion protocol. Except for $CaCl_2$, all components were diluted in Milli-Q water and autoclaved. A stock of 0.5 M $CaCl_2$ was sterilized by filtration (0.22 μm pore diameter membrane) and an appropriate aliquot was added to sterile Buffers II and III.

Buffer components	Concentration
**Buffer I**, pH 6.1	
Citric Acid	14 mM
Na_2HPO_4	51 mM
KCl	600 mM
EDTA dipotassium	10 mM
**Buffer II**	
PEG-3350	33% (w/v)
KCl	600 mM
$CaCl_2$	50 mM
**Buffer III**	
KCl	600 mM
$CaCl_2$	50 mM

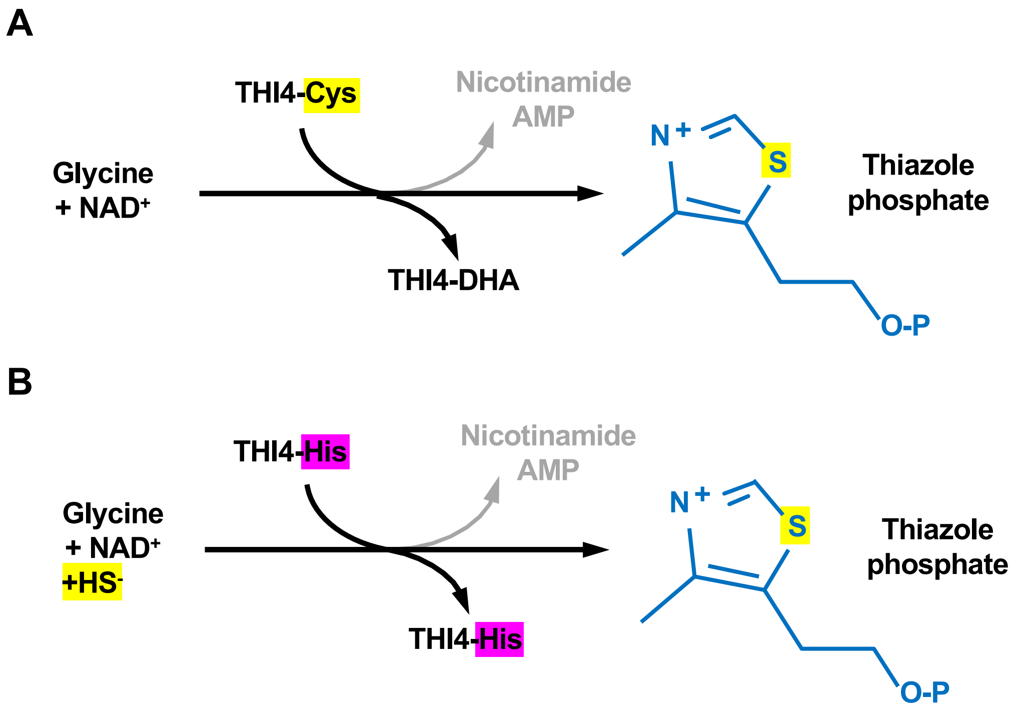

Figure S1. Reaction mechanisms of suicidal and non-suicidal THI4. In plant thiazole synthesis, THI4 uses NAD^+, glycine, and a sulfur atom from an active-site cysteine residue. In this process, this cysteine is converted to dehydroalanine (DHA) and the inactive, DHA-containing THI4 is degraded **(A).** Thermovibrio ammonificans THI4 contains a histidine in place of the active site-cysteine and is truly catalytic, using sulfide (HS^-) as the sulfur donor **(B).**

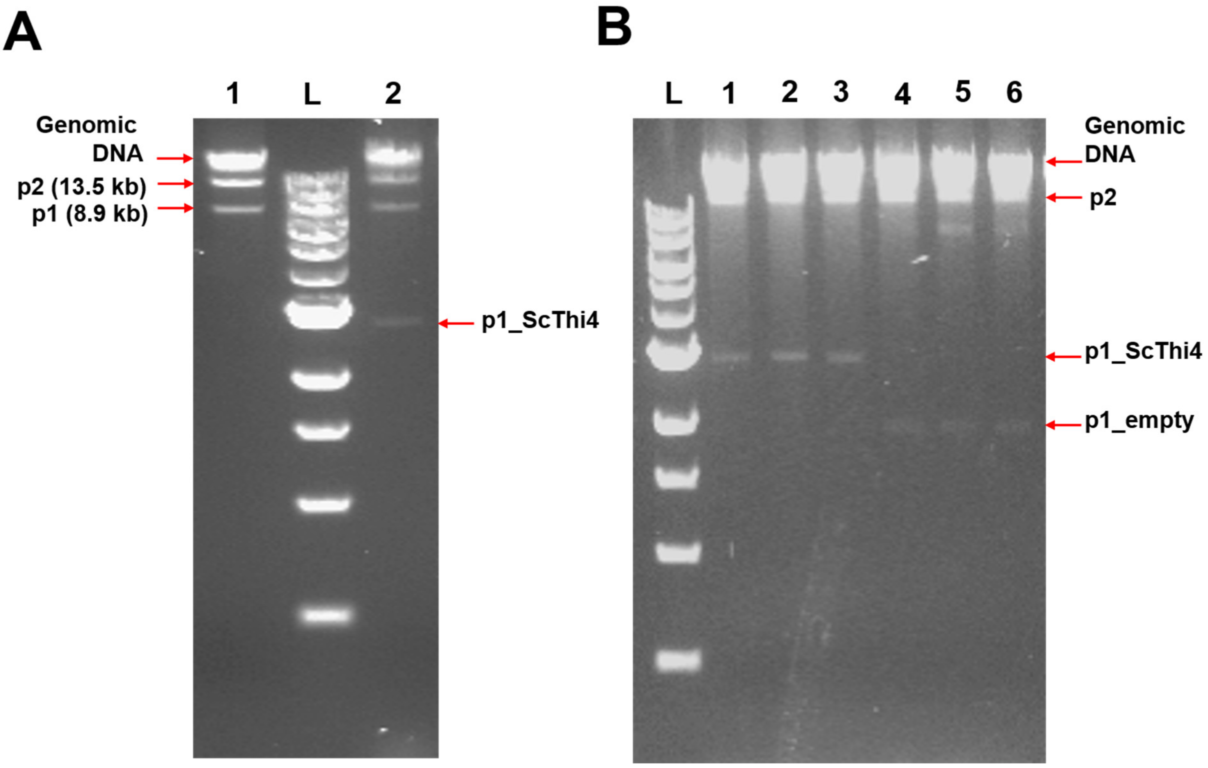

Figure S2. Gel analysis of DNA extracted from different yeast strains. DNA was separated by 1% (w/v) agarose gel. Lanes marked L contained a 1kb DNA ladder. **(A)** DNA from the GA-Y319 strain contained the wild type p1 (8.9 kb) and p2 (13.5 kb) linear plasmids (Lane 1). After transformation of GA-Y319 cells with a digested p1_ScTHI4 integration vector, an additional band corresponding to the recombinant p1_ScTHI4 (3.05 kb) was detected (Lane 2). **(B)** Screening of Δ*THI4* BY4741 clones after protoplast fusion. DNA from different clones contained the linear plasmids p1_ScTHI4 (lanes 1-3) or p1_empty (lanes 4-6).

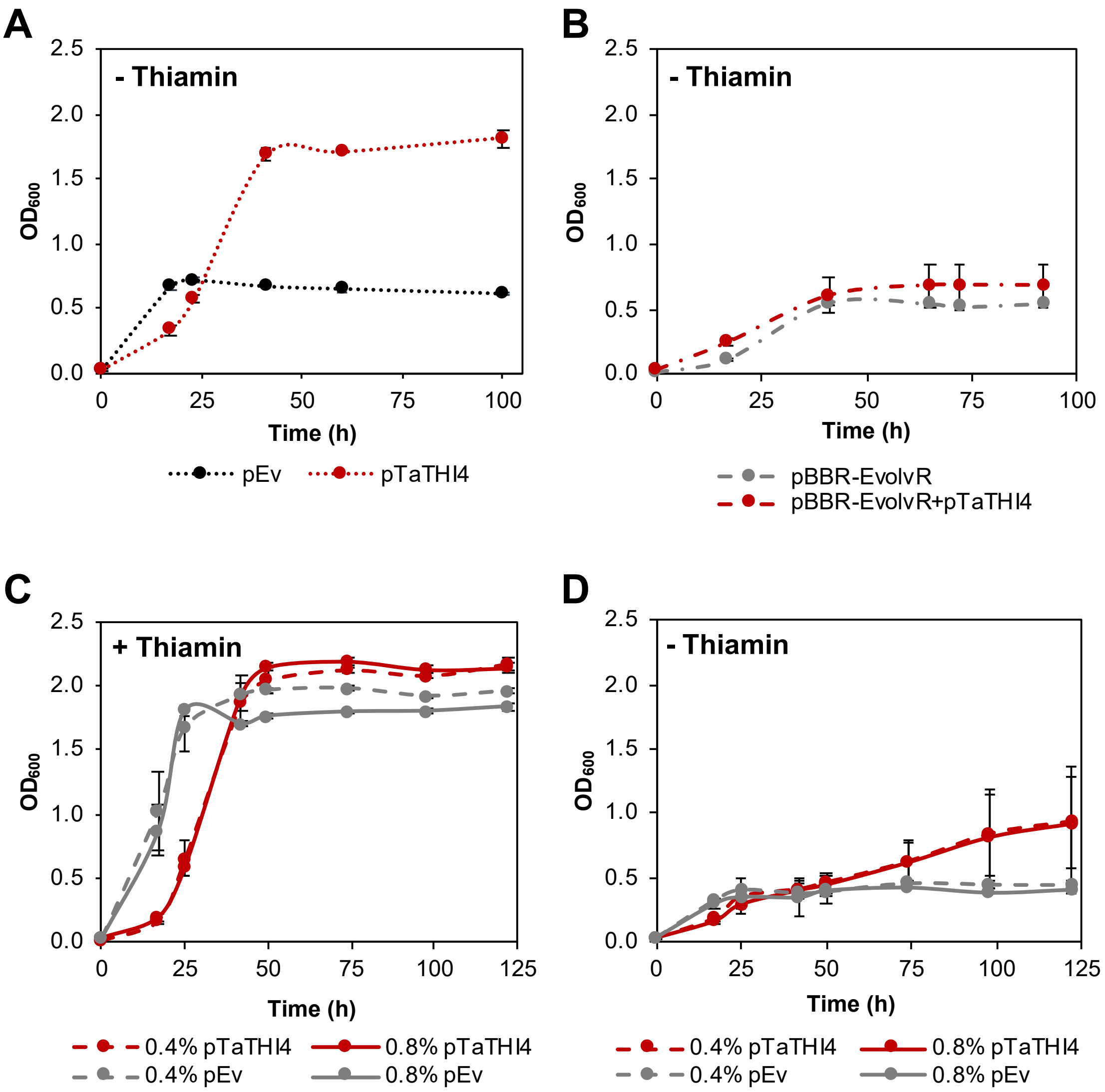

Figure S3. Troubleshooting the complementation of an *E. coli* Δ*thiG* strain by TaTHI4 using a modified EvolvR plasmid and different carbon sources. *E. coli* Δ*thiG* cells harboring the pCDFDuet-placO vector alone (pEv) or containing the TaTHI4 (pTaTHI4) gene were cultured in MOPS minimal medium containing 0.2% (w/v) glycerol, 1 mM IPTG, and 1 mM cysteine without 100 nM thiamin **(A)**. Δ*thiG* cells harboring a modified pEvolvR vector (pBBR backbone) with or without pTaTHI4 were cultured in MOPS minimal medium plus glycerol, cysteine, and IPTG as above, without 100 nM thiamin **(B)**. E. coli ΔthiG cells were cultured as in (A) but replacing glycerol with either 0.4% or 0.8% (w/v) glucose as indicated, with 100 nM thiamin **(C)** or without thiamin **(D)**. Values are means ± standard error (SE) of three independent replicates. Where no error bars appear, they are smaller than the symbol.

© 2020 by the authors. Licensee MDPI, Basel, Switzerland. This article is an open access article distributed under the terms and conditions of the Creative Commons Attribution (CC BY) license (http://creativecommons.org/licenses/by/4.0/).
